# The Vitamin E Analog Gamma-Tocotrienol (GT3) and Statins Synergistically Up-Regulate Endothelial Thrombomodulin (TM)

**DOI:** 10.3390/ijms17111937

**Published:** 2016-11-18

**Authors:** Rupak Pathak, Sanchita P. Ghosh, Daohong Zhou, Martin Hauer-Jensen

**Affiliations:** 1 Division of Radiation Health, College of Pharmacy, University of Arkansas for Medical Sciences, Little Rock, AR 72205, USA; DZhou@uams.edu (D.Z.); mhjensen@uams.edu (M.H.-J.); 2 Armed Forces Radiobiology Research Institute, Uniformed Services University of the Health Sciences, Bethesda, MD 20889, USA; sanchita.ghosh@usuhs.edu; 3 Surgical Service, Central Arkansas Veterans Healthcare System, Little Rock, AR 72205, USA

**Keywords:** gamma-tocotrienol, statins, thrombomodulin, activated protein C, endothelial cells, Kruppel-like transcription factors

## Abstract

Statins; a class of routinely prescribed cholesterol-lowering drugs; inhibit 3-hydroxy-3-methylglutaryl-coenzymeA reductase (HMGCR) and strongly induce endothelial thrombomodulin (TM); which is known to have anti-inflammatory; anti-coagulation; anti-oxidant; and radioprotective properties. However; high-dose toxicity limits the clinical use of statins. The vitamin E family member gamma-tocotrienol (GT3) also suppresses HMGCR activity and induces TM expression without causing significant adverse side effects; even at high concentrations. To investigate the synergistic effect of statins and GT3 on TM; a low dose of atorvastatin and GT3 was used to treat human primary endothelial cells. Protein-level TM expression was measured by flow cytometry. TM functional activity was determined by activated protein C (APC) generation assay. Expression of Kruppel-like factor 2 (KLF2), one of the key transcription factors of TM, was measured by quantitative reverse transcription polymerase chain reaction (qRT-PCR). TM expression increased in a dose-dependent manner after both atorvastatin and GT3 treatment. A combined treatment of a low-dose of atorvastatin and GT3 synergistically up-regulated TM expression and functional activity. Finally; atorvastatin and GT3 synergistically increased *KLF2* expression. These findings suggest that combined treatment of statins with GT3 may provide significant health benefits in treating a number of pathophysiological conditions; including inflammatory and cardiovascular diseases.

## 1. Introduction

A tightly packed monolayer of endothelial cells, called endothelium, lines all the blood vessels in the body. Endothelium regulates a number of blood functions and acts as a selective barrier between blood and tissue [1]. Thrombomodulin (TM), an endothelial surface receptor, is expressed on the luminal surface of endothelium and plays a pivotal role in maintaining an array of vascular endothelial functions [2]. TM is structurally divided into five domains (i.e., N-terminal lectin-like domain, epidermal growth factor (EGF)-like repeats domain, serine-threonine rich domain, transmembrane domain, and cytoplasmic tail), each of which has unique functional properties [3]. Studies by various groups have shown that TM alone or the TM-thrombin complex suppress inflammation, coagulation, oxidative stress, complement activation, sepsis, adhesion of immune cells to the endothelium, and radiation damage [4,5,6,7,8]. Moreover, the TM-thrombin complex transforms protein C to activated protein C (APC), which is known to have anti-inflammatory, anti-coagulant, and cytoprotective properties [4]. This suggests that up-regulation of TM expression or its functional activity may provide protection against a number of pathophysiological conditions.

Cholesterol-lowering drugs, called statins or 3-hydroxy-3-methylglutaryl-coenzymeA reductase (HMGCR) inhibitors, are potent TM inducers [9,10,11]. Studies by various groups show that multiple signaling pathways (i.e., mevalonate and nitric oxide pathway) as well as numerous transcription factors (i.e., Kruppel-like transcription factors and heat shock factor 1) play an important role in statin-mediated TM induction [9,11,12,13,14]. Moreover, statins have the ability to attenuate TM suppression in various conditions of oxidative stress [15,16,17]. Clinical studies also show that statins effectively prevent ectodomain shedding of TM molecules [18,19], an important plasma marker of endothelial injury. Because statins have vascular protective properties and cholesterol-lowering abilities, they are widely used to treat primary and secondary coronary heart disease. However, statins have other therapeutic benefits, which include increasing the number of circulating endothelial progenitor cells, attenuating vascular smooth muscle cell proliferation, inhibiting platelet aggregation, stabilizing atherosclerotic plaques, suppressing vascular inflammation and adhesion, preventing dementia, and providing protection against radiation-induced normal tissue injury [20,21,22,23]. Despite these well-documented benefits, clinical studies indicate that statins may cause significant toxicity at high doses. For example, high-dose atorvastatin treatment causes hepatic toxicity [24,25] and renal injury [26].

On the other hand, the vitamin E analog, gamma-tocotrienol (GT3), is known to induce TM expression [27] and inhibit HMGCR activity by post-translation degradation of the protein, which is independent from the mechanism of statin-mediated HMGCR inhibition [28]. Moreover, GT3 has almost no adverse effects, even at high-doses [29]. GT3 is a natural antioxidant and plays an important role in preventing cardiovascular vascular, skeletal, and gastrointestinal damage under various oxidative stress conditions, including radiation-induced oxidative stress [30,31,32,33]. However, the combined effect of statins and GT3 on TM is not known.

To overcome high-dose toxicity of statins without compromising their health benefits, particularly the TM-mediated positive health effects, we decided to use low doses of a statins and GT3 together to induce TM. We found that a combined treatment of atorvastatin and GT3 synergistically up-regulate TM expression and its functional activity in primary human endothelial cells.

## 2. Results

### 2.1. Dose-Response of Atorvastatin and GT3 on TM Expression

**Figure 1:**
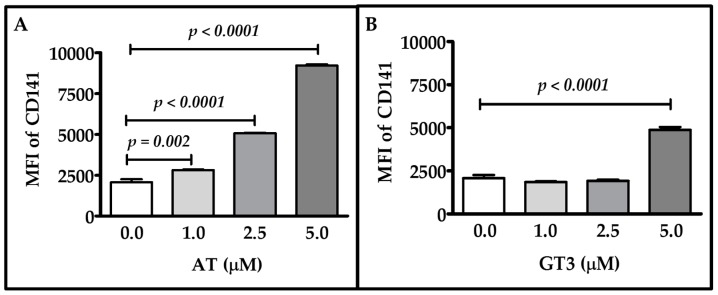
Effect of various doses of atorvastatin and gamma-tocotrienol (GT3) on thrombomodulin (TM) expression. Human umbilical vein endothelial cells (HUVECs) were either treated with vehicle or different concentrations (1, 2.5, and 5 μM) of atorvastatin (AT) (**A**) or various doses (1, 2.5, and 5 μM) of GT3 (**B**) for 24 h. TM expression was measured by flow cytometry using an anti-TM antibody, CD141 (an alternate name of TM). TM protein expression was measured as mean florescence intensity (MFI) against its respective isotype control. The data represent mean ± standard deviation (SD) of three independent experiments and each experiment consisted of three replicates per sample. Differences in TM expression between vehicle-treated and drug-treated groups were analyzed by Student’s *t*-test; *p* values less than 0.05 were considered statistically significant.

To measure the effects of atorvastatin and GT3 on TM expression, primary human umbilical vein endothelial cells (HUVECs) were treated with different doses of atorvastatin and GT3 for 24 h and TM expression was measured by flow cytometry. We observed dose-dependent increases in TM expression after atorvastatin treatment (Figure 1A). Treatment with 1, 2.5, and 5 μM of atorvastatin resulted in a 1.36-, 2.45-, and 4.46-fold increase in TM expression, respectively, as compared to the vehicle treated group. GT3 treatment at 2.5 μM did not cause a significant change in TM expression, but 5 μM GT3 treatment for 24 h caused a 2.35-fold increase in TM expression (Figure 1B).

These data clearly indicate that both atorvastatin and GT3 induce TM expression in a dose-dependent manner.

### 2.2. Atorvastatin and GT3 Synergistically Up-Regulate TM Expression

**Figure 2:**
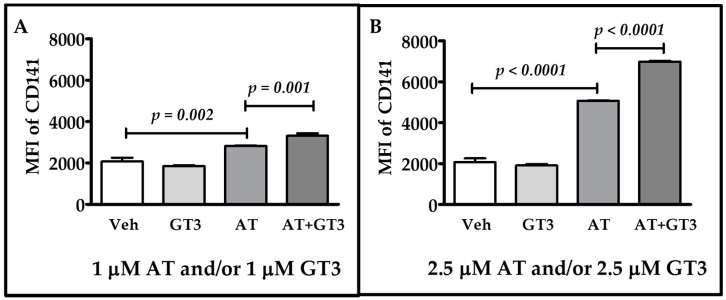
Combined effect of atorvastatin and GT3 on TM expression. TM expression as detected by flow cytometry in HUVECs pretreated with either vehicle (Veh), 1 μM GT3, 1 μM atorvastatin (AT), or 1 μM AT plus 1 μM GT3 for 24 h (**A**) and with either vehicle; 2.5 μM GT3, 2.5 μM AT, or 2.5 μM AT plus 2.5 μM GT3 for 24 h (**B**). TM expression was measured by flow cytometry using an anti-TM antibody, CD141. TM protein expression was measured as mean florescence intensity (MFI) against its respective isotype control. The data represent mean ± SD of three independent experiments and each experiment consisted of three replicates per sample. Differences in TM expression between vehicle-treated and drug-treated groups were analyzed by Student’s *t*-test; *p* values less than 0.05 were considered statistically significant.

Next, we investigated whether atorvastatin and GT3 has a synergistic effect on TM expression. To address the issue, we used low doses of atorvastatin and GT3. HUVECs were treated with 1 μM of atorvastatin, 1 μM of GT3, and 1 μM of atorvastatin plus GT3. They were also treated with 2.5 μM of atorvastatin, 2.5 μM of GT3, and 2.5 μM of atorvastatin plus 2.5 μM of GT3 for 24 h, and TM expression was measured in all groups by flow cytometry. For both the treatment strategies, we found that combined treatment resulted in significantly (*p* < 0.01) higher TM expression than singular treatment with atorvastatin or GT3 (Figure 2A,B and Figure S1). TM expression was unaltered after 1 μM and 2.5 μM of GT3 treatment, but it was increased 1.36- and 2.45-fold after 1 μM and 2.5 μM, respectively, of atorvastatin treatment. Interestingly, TM expression increased 1.6-fold after treatment with 1 μM of atorvastatin plus 1 μM GT3, and it increased 3.38-fold after treatment with 2.5 μM atorvastatin plus 2.5 μM GT3. These findings show that atorvastatin and GT3 synergistically induce TM expression in this used dose range. However, when we treated HUVECs with 5 μM atorvastatin plus 5 μM GT3, the TM expression reduced to control level at 24 h (Figure S2).

### 2.3. Atorvastatin and GT3 Synergistically Induce TM Functional Activity

**Figure 3:**
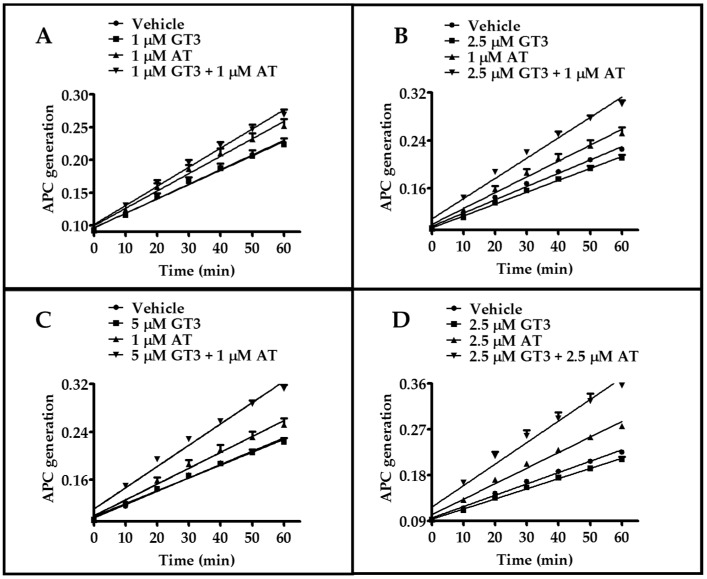
Combined effect of atorvastatin and GT3 on the functional activity of TM. Functional activity of TM as detected by activated protein C (APC) generation in HUVECs pretreated with either vehicle, 1 μM GT3, 1 μM atorvastatin (AT), and 1 μM GT3 plus 1 μM AT (**A**); or vehicle, 2.5 μM GT3, 1 μM AT, and 2.5 μM GT3 plus 1 μM AT (**B**); or vehicle, 5 μM GT3, 1 μM AT, and 5 μM GT3 plus 1 μM AT (**C**); or vehicle, 2.5 μM GT3, 2.5 μM AT, or 2.5 μM GT3 plus 2.5 μM AT for 24 h (**D**). The data are presented as mean ± SD of optical density from three independent experiments, each consisting of three to six replicates per sample.

Next, we determined whether atorvastatin and GT3 synergistically increased the functional activities of TM, which was measured by APC generation assay. HUVECs were treated with atorvastatin alone, GT3 alone, and atorvastatin plus GT3 for 24 h. We used three different doses of GT3 (1, 2.5, and 5 μM) and two doses of atorvastatin (1 and 2.5 μM). No significant change in APC generation was observed after the 1, 2.5 and 5 μM GT3 treatments. However, the 1 and 2.5 μM atorvastatin treatment for 24 h significantly (*p* < 0.001) induced APC generation when curve slopes were compared between vehicle-treated and atorvastatin-treated groups (Figure 3). APC generation was maximally induced after combined treatment with 1 μM atorvastatin plus 1 μM GT3, 1 μM atorvastatin plus 2.5 μM GT3, 1 μM atorvastatin plus 5 μM GT3, and 2.5 μM atorvastatin plus 2.5 μM GT3 treatment compared to treatment with each drug alone (Figure 3A–D), suggesting that atorvastatin and GT3 synergistically enhance the functional activity of TM as well as TM expression in this dose range.

### 2.4. Atorvastatin and GT3 Synergistically Up-Regulate KLF2 Expression

**Figure 4:**
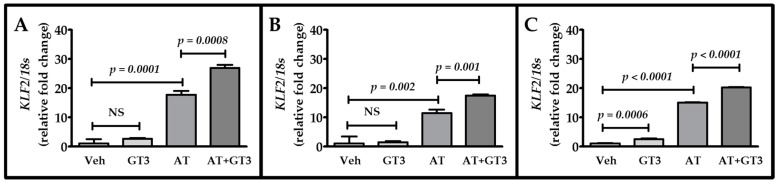
Combined effect of atorvastatin and GT3 on *KLF2* mRNA expression. Kruppel-like factor 2 (*KLF2*) expression as detected by quantitative reverse transcription polymerase chain reaction (qRT-PCR) at 6 h (**A**); 12 h (**B**); and 24 h (**C**) in HUVECs pretreated with either vehicle, 2.5 μM GT3, 2.5 μM atorvastatin (AT), or 2.5 μM AT plus 2.5 μM GT3 (AT + GT3) for 24 h. *KLF2* levels are expressed as fold change over the basal expression after normalization to 18 s. The data are presented as the mean ± SD of three independent experiments. NS, not statistically significant.

Since the transcription factor Kruppel-like factor 2 (KLF2) plays an important role in modulating TM expression, we hypothesized that atorvastatin and GT3 would also synergistically up-regulate KLF2 expression. HUVECs were treated with 2.5 μM of atorvastatin, 2.5 μM of GT3, and 2.5 μM of atorvastatin plus 2.5 μM of GT3. *KLF2* mRNA expression was measured by quantitative reverse transcription polymerase chain reaction (qRT-PCR) at three different time intervals (6, 12, and 24 h). GT3 treatment alone was not able to induce *KLF2* expression at the 6 and 12 h time points; however, there was an approximately 2.5-fold increase (*p* = 0.0006) at the 24 h time point (Figure 4A–C). On the other hand, treatment with atorvastatin alone significantly induced (*p* < 0.001) *KLF2* expression at all three time points (Figure 4A–C), but a maximum increase in *KLF2* expression was observed after combined treatment with atorvastatin and GT3, suggesting a synergistic effect of these two compounds on *KLF2* expression (Figure 4A–C).

## 3. Discussion

Elevated blood cholesterol is one of the most critical risk factors of cardiovascular disease, which is a leading cause of mortality worldwide. About 610,000 people die in the United States every year because of cardiovascular complications. Statins are the current standard of treatment for lowering cholesterol by inhibiting HMGCR through TM induction, but high doses of statins and interactions with other drugs that increase the potency of statins can cause a number of adverse effects. However, GT3 has also been shown to decrease HMGCR activity and induce TM without adverse effects. We combined low doses of atorvastatin and GT3 together to find out if the synergistic effect of this combination had a greater impact on TM induction than statins or GT3 treatment alone. To the best of our knowledge, we are the first to reveal that atorvastatin and GT3 synergistically induce the expression of endothelial TM, which has intrinsic anti-inflammatory, anti-coagulant, and antioxidant properties.

TM also plays a key role in the activation of the protein C pathway [2,3,4]. Our present study confirms that atorvastatin plus GT3 synergistically up-regulates APC generation, resulting in an increase in APC-mediated beneficial biological functions [34]. APC inactivates coagulation pathways by binding to protein S (PS), proteolytically degrading blood coagulation factors Va and VIIIa [35], and it modifies platelet function by binding with the receptors expressed on platelets [36]. APC also interacts with endothelial protein C receptor (EPCR) to exert anti-inflammatory functions [37], and when bound to EPCR, APC can activate protease-activated receptor 1 (PAR-1), another endothelial surface receptor that has cytoprotective and anti-inflammatory functions [38]. APC also inhibits neutrophil migration in an EPCR-independent manner—by interacting with β1-and β3-integrins—that modulates functions of innate immune cells [39]. Since atorvastatin plus GT3 induces endothelial TM expression, which mediates APC expression, atorvastatin plus GT3 could be a good strategy for treating a number of pathological conditions, preventing oxidative damage, treating septic patients [40], and in limiting radiation damage [8].

We found that atorvastatin induces *KLF2* expression and that GT3 also plays a key role in *KLF2* overexpression. Transcription factor KLF2, which has anti-fibrotic, anti-thrombotic, and atheroprotective properties, positively regulates the expression of a number of genes, including endothelial TM [41]. We and others, using gel shift studies and chromatin immunoprecipitation (ChIP) assay, show that KLF2 binds to GC-rich sequences, such as Sp/KLF sites in the TM promoter and enhances TM promoter activity [42,43]. Deletion studies indicate that -385-bp-Luc fragment of the TM promoter is essential for KLF2-mediated TM promoter activity [39]. Statins induce endothelial KLF2 expression by modulating KLF2 promoter activity and various signaling pathways. Sen-Banerjee et al. show that statins enhance KLF2 promoter activity in human endothelial cells via monocyte enhancer factor binding sites and thus induce KLF2 expression [41]. This statin-mediated KLF2 up-regulation in endothelial cells depends on enhancement of ERK5 kinase activity [44]. Others show that statins can induce KLF2 expression in endothelial cells by regulating the apelin/APJ signaling pathway [45]. In line with previous studies, we also observed that atorvastatin increases KLF2 expression at various time intervals. Moreover, we found that GT3 potentiates atorvastatin-dependent KLF2 over-expression, suggesting that a combined treatment of statins and GT3 could be a novel approach for inducing the beneficial effects of TM to treat a number of diseases.

## 4. Materials and Methods

### 4.1. Cell Line and Reagents

HUVECs were obtained from American Type Culture Collection (ATCC, Manassas, VA, USA). The EGM-2 BulletKit and HEPES (4-(2-hydroxyethyl)-1-piperazineethanesulfonic acid)-buffered saline solution were used to culture HUVEC cells and were purchased from Lonza (Walkersville, MD, USA). Dimethyl sulfoxide (DMSO) was obtained from ATCC (Manassas, VA, USA). Heat-inactivated fetal bovine serum (FBS) was obtained from Atlanta Biologicals (Lawrenceville, GA, USA). To dislodge adherent cells, 0.05% trypsin was obtained from HyClone (Pittsburgh, PA, USA). Anti-human CD141 antibody and immunoglobin G1 (IgG1), κ isotype control were purchased from BD Biosciences (Sparks, MD, USA). Recombinant hirudin from yeast was obtained from American Diagnostica Inc. (Stamford, CT, USA). Chromogenix S-2366 was obtained from DiaPharma Group Inc. (West Chester, OH, USA).

### 4.2. Cell Culture and Drug Treatment

HUVECs were cultured in the EGM-2 BulletKit and passaged every 2–3 days using standard aseptic techniques. HUVECs between passage numbers 2 and 7 were used for the studies. Cells from 70% to 90% confluent flasks with more than 90% viability were seeded onto culture dishes, plates, or flasks, depending on experimental requirements, prior to drug treatment. Atorvastatin stock was prepared by dissolving atorvastatin in absolute ethanol, while GT3 stock was prepared using DMSO.

### 4.3. Flow Cytometry

HUVECs were grown in 6-well plates and treated with predetermined concentrations of atorvastatin and/or GT3 for various time intervals. Cells were then trypsinized, washed twice with PBS, (phosphate buffer saline), blocked in PBS with 4% FBS for 30 min, and stained for surface thrombomodulin/CD141 molecules. Staining was achieved by incubating 100 µL of the cell suspension with phycoerythrin (PE)-labeled primary antibody in PBS with 4% FBS for 30 min. Data was acquired on an LSRII flow cytometer (BD Biosciences) and analyzed with FlowJo cytometry software from Tree Star Inc. (Ashland, OR, USA). Fluorescent labeling was measured using 10,000 cells per sample, and appropriate isotype controls were used for each experiment. Delta median fluorescence intensity (ΔMFI) was calculated by subtracting the fluorescence of the isotype from that of the drug treatment groups.

### 4.4. APC Generation Assay

HUVECs were seeded at a density of 2 × 10^4^ cells/well in 96-well plates 24 h prior to any treatment. Cells were then treated with atorvastatin and/or GT3 for 24 h. Cells of each well were washed twice with PBS and incubated with a master mix of thrombin (1 nmol/L), protein C (0.5 µmol/L), and buffer in a total volume of 60 μL for 1 h. At the end of 1 h of incubation at 37 °C, 20 μL of hirudin (0.2 unit/µL) was added to each well to neutralize the thrombin. Ten minutes after adding hirudin, 100 μL of chromogenic substrate S-2366 (0.5 mM) was added to each well, and the change in absorbance at 405 nm (millioptical density/min) was measured using a microplate reader (BioTek, Winooski, VT, USA). A standard curve with known concentrations of APC was used to determine the concentrations of APC generated in the reaction mixture.

### 4.5. Total RNA Extraction, cDNA Preparation, and qRT-PCR by Taqman Assay

HUVECs were washed twice with calcium- and magnesium-free cold PBS. Total RNA was extracted using the RNeasy Plus Mini Kit from Qiagen (Valencia, CA, USA), as per the manufacturer’s instructions. Each RNA sample was subsequently treated with TURBO™ DNase (Ambion, Grand Island, NY, USA) to remove traces of genomic DNA. RNA concentration was measured using NanoDrop™ 2000c spectrophotometers (ThermoFisher Scientific, Pittsburgh, PA, USA). A total of 2 µg of RNA was used to prepare cDNA using a high-capacity cDNA reverse transcription kit from Applied Biosystems (Carlsbad, CA, USA). qRT-PCR for different genes of interest was carried out using an ABI Prism 7000 Sequence Detection System (Applied Biosystems). Standard real-time PCR (50 °C for 2 min and 95 °C for 10 min, followed by 50 cycles of 95 °C for 15 s and 60 °C for 60 s) with the TaqMan 2X Universal PCR master mix from Applied Biosystems was used to quantitatively measure mRNA. Gene expression assays were performed using the inventoried TaqMan assay from Applied Biosystems with 6-carboxyfluorescein (FAM)-labeled probes. Expression of each gene of interest was normalized to 18 s ribosomal RNA for each of the samples. The relative mRNA expression was calculated using the relative difference in threshold cycles (CT) of target gene and the reference gene or CT (2^−ΔΔ*C*t^) method.

### 4.6. Statistical Analysis

Results are expressed as mean ± standard deviation (SD). Data were analyzed using Prism software (version 4.0; GraphPad, La Jolla, CA, USA). For experiments in which only one parameter was under consideration, the statistical uncertainty between two groups was determined by unpaired Student’s *t*-test. One-way analysis of variance (ANOVA) was conducted and was followed by Tukey’s multiple comparison. A probability value of *p* < 0.05 was considered statistically significant.

## 5. Conclusions

**Figure 5:**
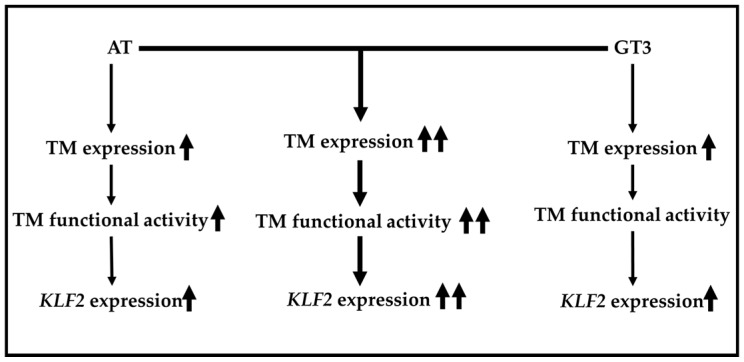
A schematic representation of the combined effect of atorvastatin and GT3 on TM expression and function as well as *KLF2* expression. A more pronounced effect on TM expression and function and *KLF2* expression was observed after combined treatment with atorvastatin (AT) and GT3 (middle column, indicated by two arrows) than after treatment with each drug alone (left and right panel, represented by single arrow).

Our findings clearly demonstrate that atorvastatin and GT3 can both induce TM expression in a dose-dependent manner. Moreover, atorvastatin and GT3 can synergistically induce TM expression and functional activity. Finally, the synergistic effect of atorvastatin and GT3 on *KLF2* expression is also evident. A schematic representation of our current findings is shown in Figure 5. This study has important implications because it offers a novel approach to induce endothelial TM and *KLF2* by combining a low-dose treatment of atorvastatin and GT3. Induction of TM and *KLF2* may be a promising strategy to treat inflammatory, coagulation, septic, and cardiovascular disorders. A more detailed understanding of the underlying molecular mechanisms of endothelial TM and *KLF2* up-regulation as a result of treatment with atorvastatin and GT3 is now needed, and further studies are required to determine whether atorvastatin and GT3 can synergistically induce TM and *KLF2* in higher eukaryotic animals.

## Supplementary Materials

Supplementary materials can be found at www.mdpi.com/1422-0067/17/11/1937/s1.
