# Supplementary material for: The Vitamin E Analog Gamma-Tocotrienol (GT3) and Statins Synergistically Up-Regulate Endothelial Thrombomodulin (TM)
Source: Int J Mol Sci. 2016 Nov 18;17(11):1937. doi: 10.3390/ijms17111937 (PMC5133932; doi:10.3390/ijms17111937)
Supplement: Supplementary file 1 [file ijms-17-01937-s001.pdf]

Supplementary Materials: The Vitamin E Analog γ-Tocotrienol (GT3) and Statins Synergistically Up-Regulate Endothelial Thrombomodulin (TM)

Rupak Pathak, Sanchita P. Ghosh, Daohong Zhou and Martin Hauer-Jensen

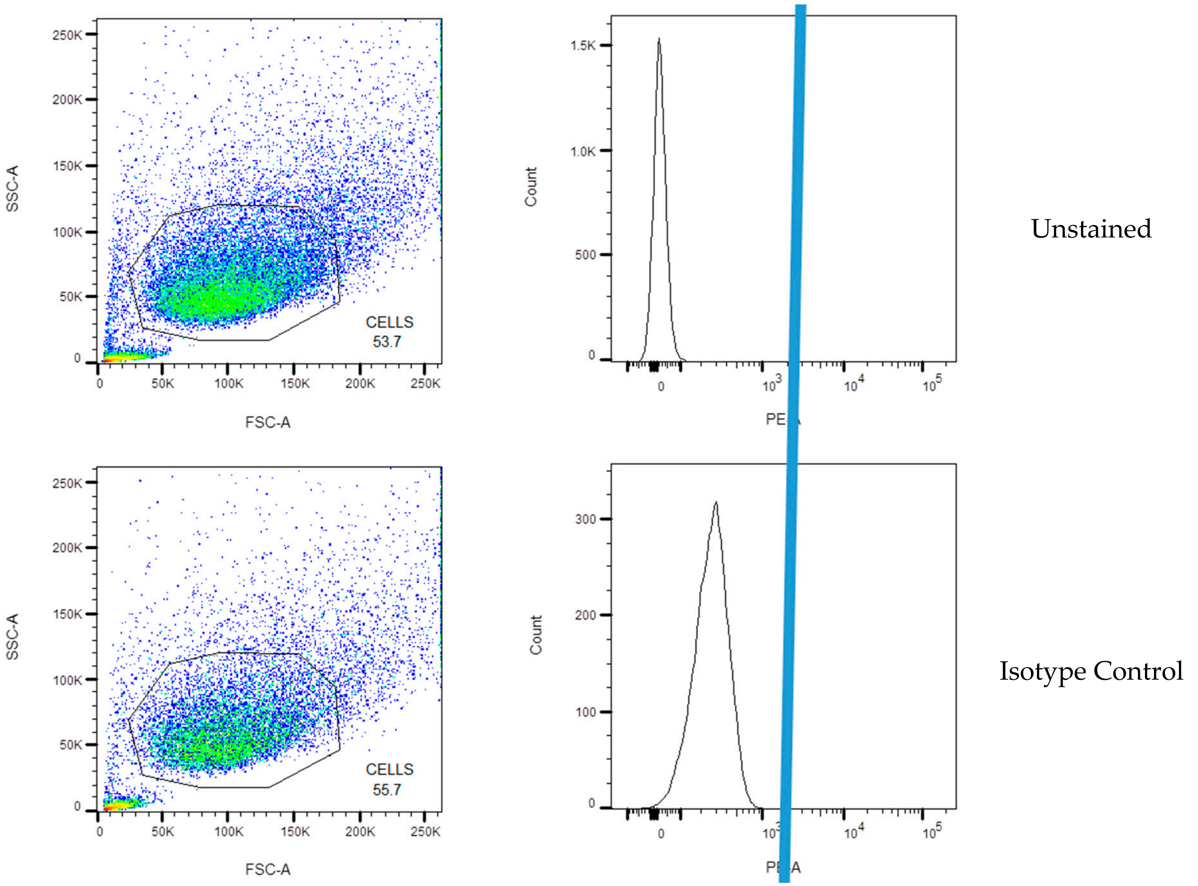

Figure S1. *Cont.*

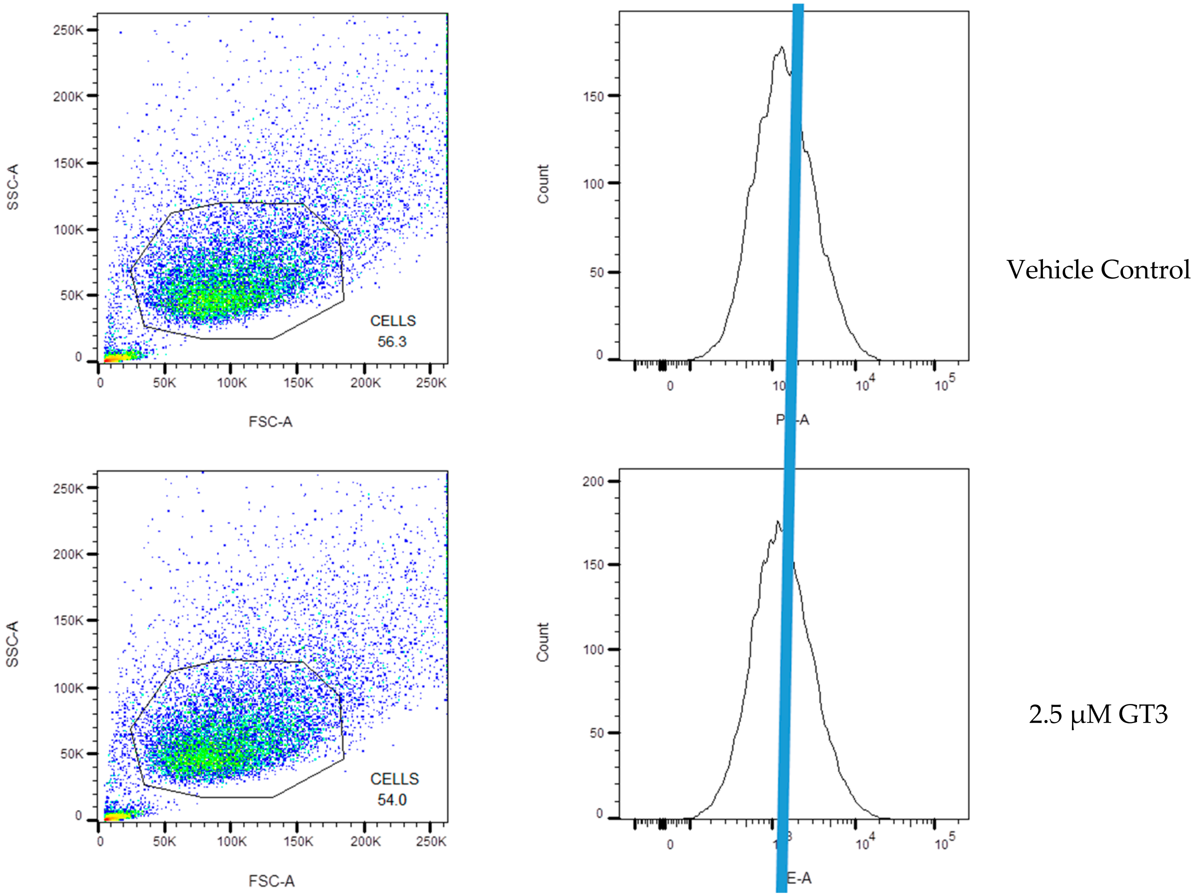

Figure S1. *Cont*.

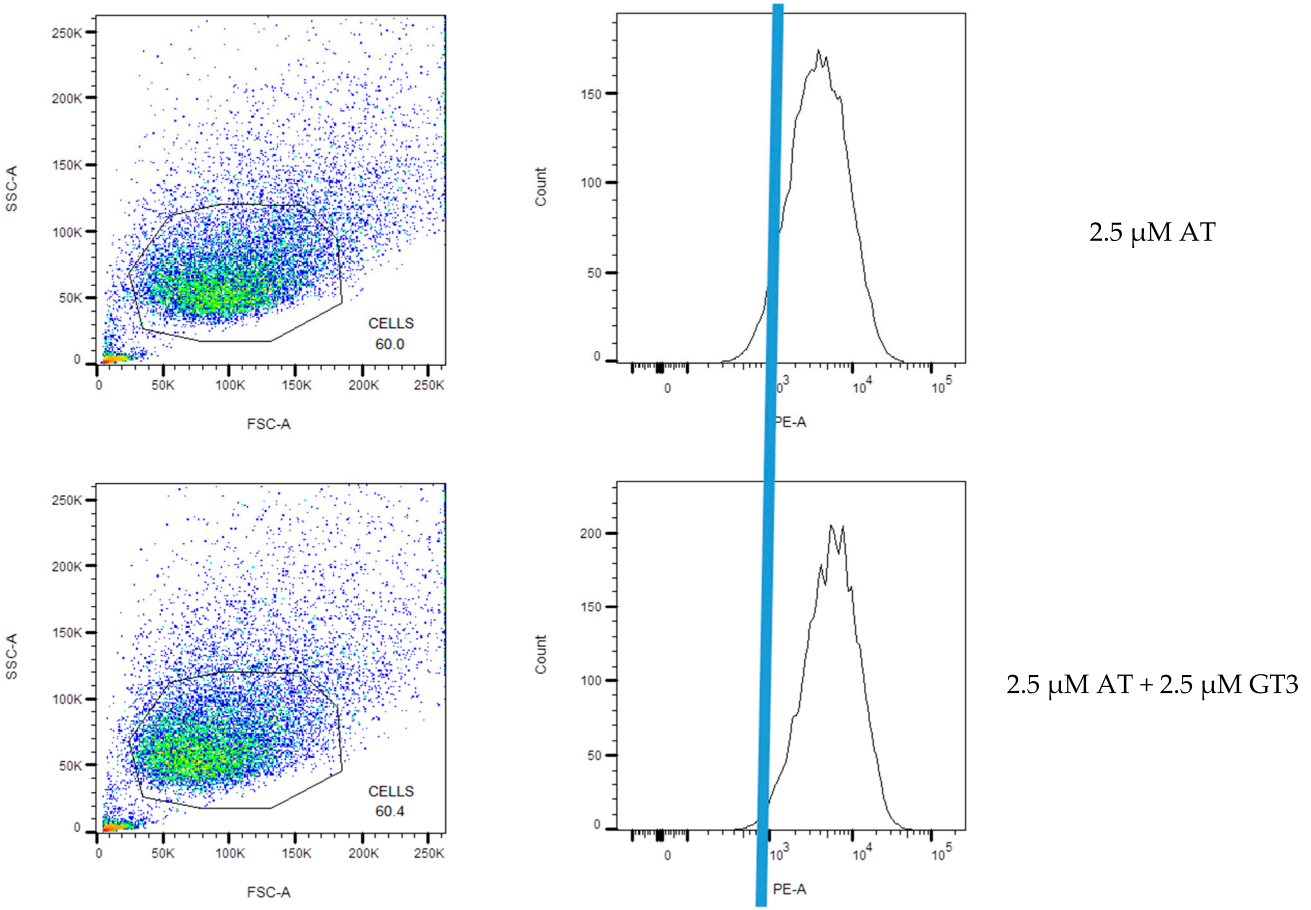

Figure S1. Combined effect of atorvastatin (AT) and γ-tocotrienol (GT3) on thrombomodulin (TM) expression. FSC-A, forward scattered area, SSC-A, side scattered area. Black circle representing the gated area.

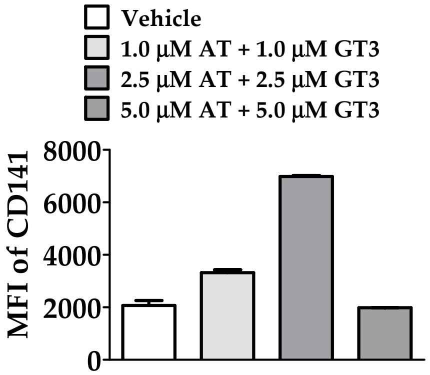

Figure S2. Synergistic effect of Atorvastatin and GT3 on TM expression.
